# Assessment of near visual acuity in 0–13 year olds with normal and low vision: a systematic review

**DOI:** 10.1186/s12886-016-0386-y

**Published:** 2016-12-08

**Authors:** Bianca Huurneman, F. Nienke Boonstra

**Affiliations:** 1 Department Cognitive Neuroscience, Radboud University Medical Center, Donders Institute for Brain, Cognition and Behaviour, Nijmegen, The Netherlands; 2 Bartiméus, Institute for the Visually Impaired, Zeist, The Netherlands

## Abstract

**Background:**

The inclusion for rehabilitation of visually impaired children is partly based on the measurement of near vision, but guidelines for near visual acuity assessment are currently lacking. The twofold purpose of this systematic review was to: (i) provide an overview of the impact of the chart design on near visual acuity measured, and (ii) determine the method of choice for near vision assessments in children of different developmental ages.

**Methods:**

A literature search was conducted by using the following electronic databases: PubMed, Cochrane Library, and EMBASE. The last search was run on March 26th 2016. Additional studies were identified by contacting experts and searching for relevant articles in reference lists of included studies. Search terms were: vision test(s), vision assessment(s), visual acuity, chart(s) and near.

**Results:**

For children aged 0–3 years the golden standard is still the preferential looking procedure. Norms are available for this procedure for 6–36 month old children. For 4–7 year olds, we recommend using the LEA symbols, because these symbols have been properly validated and can be used in preliterate children. Responses can be verbal or by matching the target symbol. In children aged 8–13 years, the recommended method is the ETDRS letter chart, because letter acuity is more predictive for functional vision and reading than symbol acuity. In 8–13 year olds, letter acuity is 0.1–0.2 logMAR poorer than symbol acuity.

**Conclusions:**

Chart design, viewing distance, and threshold choice have a serious impact on near visual acuity measurements. Near visual acuity measured with symbols is lower than near visual acuity measured with gratings, and near visual acuity measured with letters is lower than near visual acuity measured with symbols. Viewing distance, chart used, and letter spacing should be adapted to the child’s development and reported in order to allow comparisons between measurements.

## Background

Near visual acuity measurements are an important part of general vision assessment. Our ability to see small details up close can be measured with near vision charts. One problem in near visual acuity assessment is that there are numerous charts available and the number is consistently growing. The measurement of near visual acuity in children is not yet part of the routine ophthalmological examination. It is time consuming and requires special skills because children are eager to shorten the viewing distance which complicates measurement of near visual acuity. Sensitivity between near visual acuity charts varies largely and has a major impact on measurement of visual acuity. It is important that clinicians are aware of these differences. The inclusion for rehabilitation of children with visual impairment (VI) is based on their near visual acuity. The outcome of near visual acuity assessment thus has personal as well as economic consequences. There is broad consensus among researchers that chart design and reading distance have an impact on near visual acuity scores, but a systematic overview of the results collected with different near vision charts is currently lacking. Reproducibility of near visual acuity measurements depends on the age and co-operation of the child and the method used. The choice for a method should be based on the developmental level of the child. This systematic review will provide guidelines for near visual acuity measurements for 0-3 year old children with normal visual acuity and low vision.

The measurement of near visual acuity is dependent on a number of factors, such as distance visual acuity, accommodation capability, and near vision correction [1]. The amplitude of accommodation in school-age children is about 15 dioptres indicating that they can see details up close at 6.7 cm [2]. However, accommodation accuracy is reported to be less precise in infants [3] and at smaller reading distances [4]. Because accommodation accuracy is dependent on viewing distance, it is important to adopt a fixed viewing distance during near vision assessments. The American Academy of Ophthalmology underlines the importance and clinical relevance of near vision testing [5]. Near vision testing is indicated for patients that come to the clinic for refractive surgery evaluation, or routine examination involving refraction, particularly for individuals aged 40 years and above, and patients with symptoms at near such as blurred vision or discomfort. The American Academy of Ophthalmology prescribes that near vision should be measured at 14 to 16 inches (35–40 cm) or at the patient’s preferred reading distance. Ideally, the patient is tested under corrected and uncorrected circumstances at an appropriate distance as determined by the patient’s needs. The viewing distance recommended is 40 cm for easy comparison between near and distance visual acuity, because at this distance the influence of accommodation is minimal. All these general recommendations concerning distance apply for near vision assessments in adults, however when testing near visual acuity in children, viewing distance is not the only factor that should be taken into account. In addition, it is of crucial importance that age appropriate and validated stimuli are used [6]. Another important factor that should be considered is response manner. A measurement of near visual acuity in children with reduced vision is particularly important, because low vision at near is an indication for rehabilitation and it can serve as a reference value for the letter size of reading material or the choice for a specific visual aid.

Near visual acuity can be measured in different ways, of which the following two are most often used in clinic: i) resolution acuity, for example tasks where the stimulus pattern (grating) has to be resolved or chosen or a gap has to be detected, and (ii) recognition acuity, for example Snellen letters or LEA symbols [7]. We will review the differences in near visual acuity obtained with resolution and recognition charts. The aim of this systematic review is to: (i) provide an overview of the impact of the chart design on the near visual acuity measured, and (ii) determine the method of choice for near vision assessments in children of different developmental ages.

## Methods

Three databases were used to select studies: PubMed, Cochrane and EMBASE. Quality of the included studies was evaluated independently by two reviewers (BH and FNB) using criteria for cross sectional studies. The last search was run on March 26th 2016. Studies that reported near visual acuity outcome measures were included if they met four inclusion criteria: (1) a cross-sectional or observational design was used, (2) the study included 0–13 year old children with normal development and normal vision, and/or 0–13 year old children with normal development and low vision, (3) the study included children without mental impairments, and (4) the study was published in an English peer-reviewed journal. The search was developed by an experienced clinical librarian together with the first author of this article. After selecting all possible studies with predefined search terms (see search strategy in Table 1), a validated child filter with high sensitivity was applied to select child studies [8].

**Table 1:** Search history in Pubmed

Search	Query	Items
#2	Search (((((Infant[MeSH] OR Infant* OR infancy OR Newborn* OR Baby* OR Babies OR Neonat* OR Preterm* OR Prematur* OR Postmatur* OR Child[MeSH] OR Child* OR Schoolchild* OR School age* OR Preschool* OR Kid OR kids OR Toddler* OR Adolescent[MeSH] OR Adoles* OR Teen* OR Boy* OR Girl* OR Minors[MeSH] OR Minors* OR Puberty[MeSH] OR Pubert* OR Pubescen* OR Prepubescen* OR Pediatrics[MeSH] OR Pediatric* OR Paediatric* OR Peadiatric* OR Schools[MeSH] OR Nursery school* OR Kindergar* OR Primary school* OR Secondary school* OR Elementary school* OR High school* OR Highschool*)))) AND #1)	275
#1	Search ((((((“Vision Tests”[Mesh:NoExp]) OR (vision test[tiab] OR vision tests[tiab] OR (testing[tiab] AND vision[tiab]) OR assessment[tiab] OR chart[tiab] OR charts[tiab]))) AND ((“Visual Acuity”[Mesh]) OR ((visual[tiab] OR vision[tiab]) AND acuity[tiab])))) AND near[tiab])	801

### Study selection

Study eligibility based on inspection of titles and abstracts was performed by the two authors (BH and FNB), using the inclusion criteria presented in Table 2. All stages of study selection, data extraction, and quality assessment were also performed by these two independent reviewers (BH and FNB). Disagreements during selection were solved by application of inclusion criteria, and reaching consensus after discussion.

**Table 2:** Inclusion criteria

Population	Children with normal vision 0–13 years
	Children with low vision 0–13 years
Intervention	Cross sectional studies
	Observational studies
Comparison	Near versus distance visual acuity
	Different near visual acuity measures
Outcome measures	Near visual acuity
	Near and distance visual acuity

### Inclusion criteria

Included quantitative studies had to adhere to the following criteria: 1) report near visual acuity scores in one of our two target groups, 2) (lowest inclusion) age of children should be between 0–13 years, and 3) the study has a cross sectional or observational design. In order to increase data collection we also searched for articles by inspecting reference lists of the included studies. Screening studies were included, but studies only involving children with amblyopia were excluded from this review since the focus is explicitly on children with normal vision and children with low vision. Children with refractive errors were included, since they can attain normal visual acuity when corrected properly.

### Data extraction and quality assessment

Quality of the included studies was evaluated independently by two reviewers (BH and FNB) using criteria for cross sectional and case-control studies. Information for evaluation of the included studies was: number of participants, method used, a clear outcome definition (in this case near visual acuity scores), and results (reporting confidence intervals and thresholds in case they were presented). The first author extracted the data and contacted authors of identified studies for additional data if necessary.

### Statistical analysis

Because of the scarcity of studies providing quantitative data about near vision in children and the wide range of vision tests used to measure near vision we report results in a narrative rather than a quantitative manner.

## Results

### Results of search and selection process

**Fig. 1:**
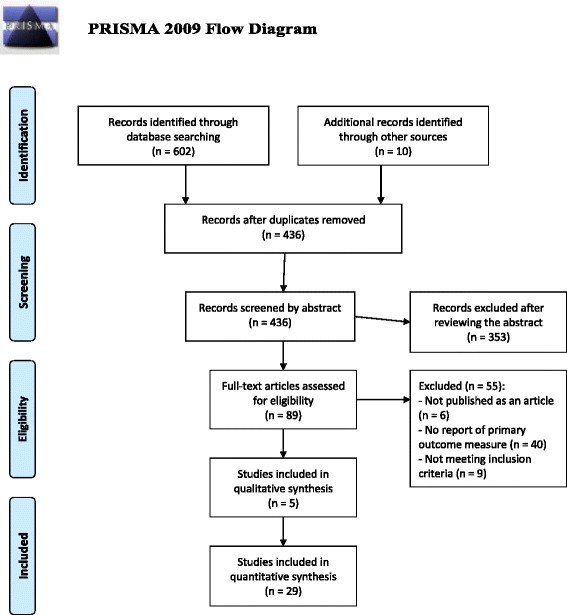
PRISMA Flow diagram

Our search in the three electronic databases provided a total of 602 citations. After removal of duplicates there were 436 studies left. After screening these studies on their titles and abstracts the number of studies for inclusion was 89. The next step was to screen full-text versions of these articles for eligibility. Fifty-five studies were excluded because they did not contain the primary outcome measure (*n* = 40), they were not published as an article (*n* = 6), or because they included the wrong population (*n* = 9). After doing this, 34 studies remained. Of the included studies, 29 were quantitative studies, and 5 were qualitative studies. See PRISMA flow chart Fig. 1.

### Description of included studies

Thirty-four studies were included. For the 0–3 year old group, 2 qualitative [9, 10], and 10 quantitative studies were included [11–20]. Of these 12 studies, only one study reported near visual acuities in children with VI [15]. For the 4–7 year old group, 12 studies were found in which near vision was measured in children with NV, and 3 studies were found for children with VI. For the 8–13 year old group, 10 studies were included which measured near vision in children with NV, and 2 studies which reported near vision in children with VI.

#### Near visual acuity assessments in 0–3 year olds

Because near visual acuity is usually measured at 40 cm or less we also included studies in which grating acuity was measured at a distance of 38 cm. Gratings are the most frequently used stimuli to measure visual acuity in infants.

First we will describe the results from two reviews on visual acuity assessments in infants. In the first review by Velma Dobson two ways to measure visual acuity in infants are described: (i) detection of the optokinetic nystagmus (OKN), and (ii) the use of preferential looking techniques [9]. The OKN is defined as ‘an ocular response elicited by the movement of a repetitious stimulus across the patient’s field’. When an OKN occurs, the eyes follow the movement of the stimulus in slow pursuit until a saccade has to be made after which smooth pursuit is visible again. Visual acuity can be assessed by manipulating stripe widths and is expressed as the smallest stripe width that can be resolved reliably by the visual system. Dobson reviewed 7 studies in which the OKN was evaluated to assess visual acuity in infants. There was good agreement across different studies concerning the mean and variability of acuity of infants. However, Dobson also mentioned that the OKN procedure has several disadvantages: (i) stimulus imperfections can elicit an OKN (so these should not be present), (ii) the judgement of the presence or absence of an OKN can be difficult for the observer, (iii) it can be challenging to keep an infant interested when using a drum, as is often the case in the clinic, because the stimulus only covers a small part of the visual field, and (iv) variations in distance between stripes of the drum can easily occur and variations in attention or acuity as a function of viewing distance can affect acuity estimates.

Another method that can be used to measure visual acuity in infants is preferential looking. Preferential looking is a procedure based on the observation that infants have a greater tendency to fixate a pattern than a homogeneous field. Position and location of the stripes is varied on each trial and acuity is, like for the OKN, expressed as the smallest stripe width the infant can see. Among preferential looking measurements there was also a good amount of agreement across research groups with regards to the mean acuity of infants; values fell within one octave of each other. In the ‘80s there was already a trend for preferential looking to be used more frequently than OKN for laboratory measurements. Factors favouring preferential looking above the OKN method are: stimuli are easier to produce, and forced choice preferential looking (FPL) is less subjective than OKN. Judging the presence of an OKN can be difficult and in FPL proportions correct are scored and a psychometric curve can be plotted. Dobson provides three criteria for tests to be clinically useful: efficiency (measurements should not take too long), facility (tests should be easy to adopt), and validity (infants with poor acuity should do poorly on the test).

The second qualitative review was written 3 years later by Davida Teller [10]. Teller describes three problems with the use of preferential looking techniques: (i) testing can only be done by trained personnel, (ii) the lack of knowledge about usefulness in clinical populations, and (iii) the inherent statistical limitations of the available techniques which put the technique at the “outer margin of efficiency for clinical routine use”. When looking back at Teller’s review, one thing that is particularly striking in this more than 30 year old paper is that the following picture was drawn for the future to come: “… the quality and intensity of the infant’s staring behaviour on each trial contains more information than one gets out of the single left-right judgement imposed by the forced choice method. Thus, one might be able to use the stimuli and approach of FPL, but abandon some of its formal aspects, and simply use a few responses to grating targets as a basis of a subjective clinical judgement of the infant’s acuity”. The second idea that she presented was the development of new equipment to measure visual acuity in the future, based on video displays run by computers. She mentioned that before these techniques could be used for clinical evaluation, the new equipment should have population norms, present proper population distributions, and realistic estimates of standard errors in the measurements.

**Fig. 2:**
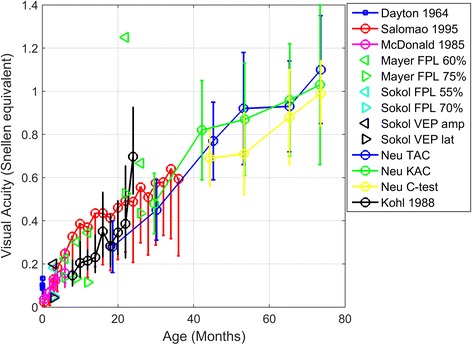
Mean visual acuity scores for newborns, infants and young children. Error bars of the Salomao study indicate lower bounds (95%). Error bars ± 1 SD

In addition to the review studies, 10 quantitative studies were found, spanning a total time period of 51 years. One study measured visual acuity in newborns by detecting an OKN [11]. The other 9 studies used different variants of preferential looking procedures: (i) a combination between forced preferential looking and operant reinforcement techniques, (ii) 2 studies using FPL solely, and (iii) 7 studies using the Teller Acuity Card (TAC) procedure. As can be seen in Fig. 2, the FPL tends to provide visual acuity estimates that are below the ones collected with the TAC-procedure, especially when a 70 or 75% threshold is used.

### Impact method on VA measured

Table 3 presents the design, participant characteristics, method and outcome of the included studies. Figure 2 provides visual acuity estimates collected with different procedures. The first main message that can be extracted from Fig. 2 is that the 70 and 75% FPL thresholds seem to be too high for young children, resulting in an underestimation of their perceptual abilities [12, 14]. A recent study also provided evidence that the optimal threshold for young children is often below 50% correct, because of high lapse rates and relatively lower rates of guessing correctly in infants [20]. Another important aspect of near vision assessment in young children (aged 2–6 years) is that optotype tests are more effective test in detecting uncorrected refractive errors than preferential looking paradigms. A disadvantage of the C-test is that it cannot always be used successfully in young children (testability is 87% in children aged 26–72 months) [18].

**Table 3:** Type of study and outcome for quantitative studies on near visual acuity in 0–3 year olds

Reference	Type of study	Number of participants, group (and age).	Method	Outcome
Dayton 1964 [11]	Cross sectional	*n* = 39 Group = NV Age = 0–8 days	Procedure: Binocular OKN + electro-oculography Range stimuli: 20/150, 20/290, 20/440 (5 mm/s) Threshold used: 50% Viewing distance: 37 cm from center % Successful: 45%	See Fig. 2.
Mayer 1980 [12]	Cross sectional	*n* = 6 Group = NV Age = 6–24 months	Procedure: Binocular Operant Preferential Looking (OPL) Range stimuli: 20/5 to 20/640 (octave steps), stimuli were selected for each child 4/5 widths per child. Threshold used: 60% + 75% (psychometric curve) Viewing distance: 57 ± 3 cm from target % Successful: 100% Test duration: 45–60 minutes	See Fig. 2.
McDonald 1985 [13]	Cross sectional	*n* = 8 Group = NV Age = 4w-6 months	Procedure: Binocular acuity card procedure Range stimuli: 0.4–24 cpd (octave steps) Threshold used: Finest grating that infant can see Viewing distance: 36 ± 3 cm from center % Successful: 100% Test duration: 3–5 minutes	See Fig. 2.
Sokol 1985 [14]	Cross sectional	*n* = 26 Group = NV Age = 3 months	Procedure: Binocular VEP and FPL Range stimuli: 0.31,0.62,1.25, 2.5 cpd Threshold used: 55% and 70% FPL and VEP lat + amp % successful: 65%	See Fig. 2.
Preston 1987 [15]	Cross sectional	*n* = 20 Group = VI Age = 2–8 months	Procedure: Mono- and binocular acuity card procedure and FPL Range stimuli: 0.2–2.0 cpd (half octave steps) Threshold used: Finest grating that infant can see Viewing distance: 33 ± 3 cm %Successful: 100% (mono- and binocular) 83% monocular FPL Test duration: 8 minutes	10/20 subjects were identified as having subnormal acuity on at least one test (binocular, left or right eye). Acuity card procedure is validated for use in patients.
Kohl 1988 [16]	Longitudinal study	*n* = 18 Group = NV Age = 12–24 months	Procedure: Mono- and binocular acuity card procedure Range stimuli: 20/25-20/3200 (octave steps) Threshold used: Finest grating that infant can see. Viewing distance: 34 cm %Successful: Mean testable 12–24 month group 79.6% lower than 90% testable for the 0–12 month old group.	See Fig. 2.
Salomao 1995 [17]	Cross sectional	*n* = 646 Group = NV Age = 0–36 months	Procedure: Monocular and binocular TAC Range stimuli: 0.23–38 cpd (half-octave steps). Start card 0.44 cpd 1–6 months, 1.3 cpd 10–18 months, 2.4 cpd >20 months Threshold used: Staircase. Last card with 2 correct responses. Viewing distance: 0–6 m: 38 cm; >6 months 57 cm. % Successful: 99.3% (binocular) 96.2% (monocular) Duration: 13 min. for one binocular and two monocular measurements	See Fig. 2.
Neu 1997	Cross sectional	*n* = 210 Group = NV Age = 1–6 years	Procedure: Monocular TAC, KAC (resolution acuity) and C-test (recognition acuity) Range stimuli: TAC: 0.32–0.38c/cm (half-octave steps), KAC: 0.40–49.2 c/cm, C-test: 0.1–1.4 (decimal) Distance: TAC and KAC: 38, 55 and 84 cm; C-test: 40 cm Threshold used: TAC as above (Salomao); C-test (3/4) 75% % Successful: TAC 40% < 2y; 96% 2-4y;	See Fig. 2. C-test gives lower acuity estimates than TAC and KAC and has higher sensitivity of the C-test for detecting uncorrected refractive errors.
Jones 2014 [19]	Cross sectional	*n* = 30 Group = NV Age = 2.6–12.7 months	Procedure: Binocular validation of computerized acuity card procedure using an eye tracker (ACTIVE) Range stimuli: 0.18–12.5 cpd (KIAC), ACTIVE started at 0.36 cpd and used the same staircase procedure as TIAC Distance: 38 cm (KIAC) and 84 cm (ACTIVE) Threshold used: ACTIVE 33.3% (up2-down 1 staircase) % Successful: 100%	ACTIVE acuities fell within the 90% range of TAC acuity norms. In 101 s a reliable VA could be obtained. Test-retest data showed difference of 0.04 octaves, which is very small. Larger study needed to collect norms.
Jones 2015 [20]	Cross sectional	*n* = 55 Group = NV Age = 2.6–12.7 months	Procedure: Binocular ACTIVE (see above) Range stimuli: see above Threshold used: 33.3% and 70.7%	This paper stresses the importance of using a low threshold (<50%) in infants or max correct response to assess perceptual sensitivity.

### Method of choice

The golden standard for testing near visual acuity in 0–36 month old children is the TAC, providing norm scores across the whole age range and validated for children with NV [17] and low vision [15]. For the TAC, a left/right 2 alternative forced choice procedure is used with a 50% guess rate. During TAC assessments, the observer reports whether the infant directed his/her gaze towards the side with a grating pattern. A critique on the TAC is that norm scores have lower boundaries that are not representative for normal vision; they should be higher) [17]. These low boundaries affect the test’s sensitivity to pick up near vision problems. The second critique is that the visual acuity that is measured relies heavily upon the subjective judgement of an observer [19].

More than 30 years after Teller’s review, there are now developments towards a computerized version of the TAC using eye-trackers to measure gaze direction. This new method offers a more objective and standardized way of measuring visual acuity in children than the TAC, because TAC acuities rely solely on a subjective report of whether or not the child’s gaze was directed at a target. A computerized version of the TAC has several advantages: (i) it is fully automated and scoring does not rely on an observer, and (ii) it has control over key parameters that, ideally, should be standardized (e.g. luminance, presentation distance, and location of the stimulus in the visual field). However, it also has disadvantages, because certain eyes are not easy to track, for instance because of nystagmus or iris transillumination. So, a computerized application of the TAC might be very useful, but since eye-tracking is not always an option, it is likely that TAC cards will still be used in the future.

#### Near visual acuity assessments in 4–7 year olds

**Fig. 3:**
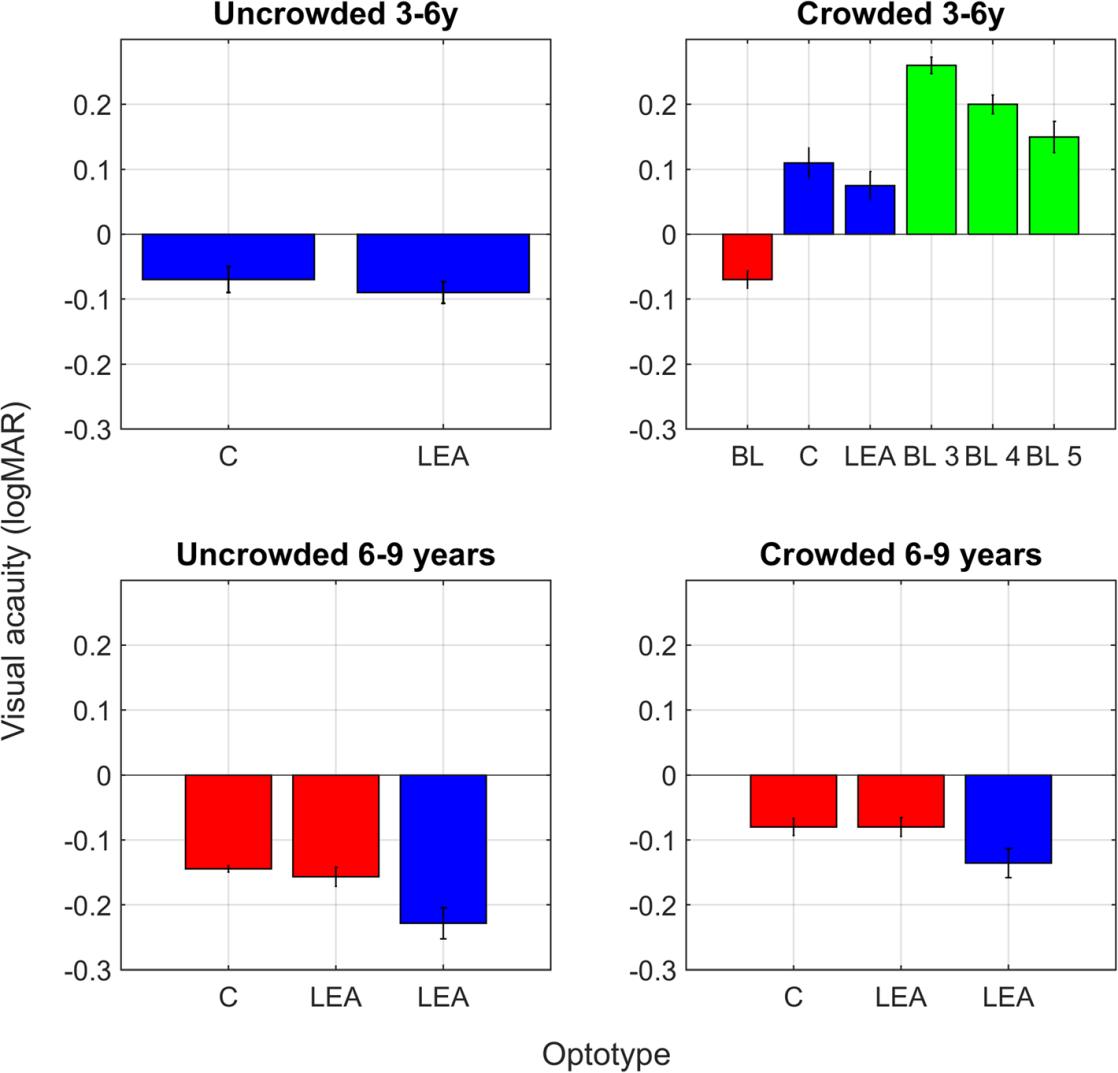
Near visual acuities in children with normal vision (NV) aged 3–9 years. In the upper panels, blue bars represent data from the Huurneman 2012 study, red bars represent data from the Dowdeswell study, and green bars represent data from the Huang study. In the lower panels, red bars represent data from Huurneman in 2012 and blue bars represent data from the Huurneman 2013 study. Error bars ± 1 standard error of the mean (sem)

**Fig. 4:**
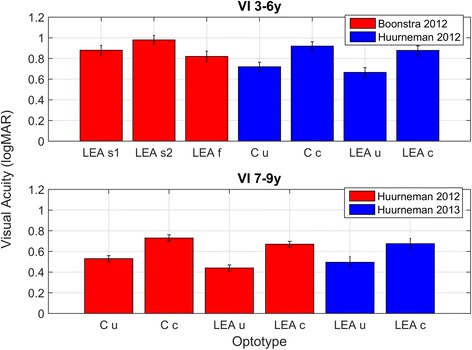
Near visual acuities in children with visual impairment (VI) aged 3–9 years. LEA s1 = LEA line chart with 1× optotype spacing at self-chosen viewing distance. LEA s2 = LEA line chart with 0.5× optotype spacing at self-chosen viewing distance, and LEA f = LEA line chart with 0.5× optotype spacing at fixed distance of 40 cm. Note the difference of 0.16 logMAR between LEA s2 and LEA f which is due to differences in viewing distance. C u = Landolt C test with absolute spacing of ≥ 30 arc min, C c = Landolt C test with 2.6 arc min spacing. LEA u = Lea uncrowded symbols with absolute optotype spacing of ≥ 30 arc min. LEA c = Lea crowded symbols with 2.6 arc min optotype spacing. Error bars ± sem

A total of 12 studies were included [21–32]. Three of these studies were included for qualitative purposes [24, 25, 32]. Table 4 presents the design, participant characteristics, method and outcome of the included studies. Figure 3 displays the results of four studies in children with NV in which means and standard deviations were provided. Figure 4 displays the results of 3 studies in which near visual acuity means and standard deviations were provided for a sample of children with VI.

**Table 4:** Type of study and outcome for quantitative studies on near visual acuity in 4–7 year olds

Reference	Type of study	Number of participants, group (and age).	Method	Outcome
Heller 1974 [25]	Observational	*n* = 40 Group = NV Age = 2 ½–6 years	Test: Binocular near point acuity test card Optotype spacing: 2× letter size (edge-to-edge) Distance: 33 cm Response: Verbal.	No validation against existing charts, just determination whether 20/20 acuity was achievable with the chart. This was the case.
Ismail 1981 [32]	Cross sectional	*n* = 4239 Group = NV Age =5.57 years (mean)	Test: Mono- and binocular near and distance (Sheridan-Gardiner single letter test) Optotype spacing: not specified Distance: not specified. Threshold: not specified. Response: not specified.	N5 and N6* (20/20 or 20/30) were taken as normal. Children with vision of 20/40 or lower were referred. 99.2% achieved normal vision. In the 12 children with N8 or worse low vision aids were considered.
Hohmann 1982 [31]	Cross sectional	*n* = 62 Group = NV Age = 6–12 years	Test: Binocular Landolt C-test Range: 0.1–1.4 (decimal acuity) Spacing: 2.6′ and 17.2′ Distance: 40 cm (and 6 m) Threshold: 88–94% Response: Verbal or matching	The majority of subjects had vision of 1.4 (decimal). Maximum acuity uncrowded optotypes at 7 years and crowded optotypes at around 10 years.
Dowdeswell 1995 [23]	Cross sectional	*n* = 68 Group = NV Age = 5;2–7;6 years	Test: Monocular Bailey-Lovie chart at 0.3 and 6 m Range: 0.1–2.0 Spacing: 1× optotype size Distance: 30 cm and 6 metres Response: Not specified	See Fig. 3.
Lovie-Kitchin 2001 [30]	Cross sectional	*n* = 71 Group = low vision Age = 7–18 years	Test: Binocular near text visual acuity (reading test based on the Minnesota Low Vision Reading Test) and distance visual acuity (Bailey-Lovie chart) Spacing: not specified Distance: 10 cm and 3 metres Threshold: DVA scored per letter Response: reading	Distance vision ranged from 0.10–1.28 logMAR and near text visual acuity from 0.12–1.47 logMAR (N 1.5-N24 at 10 cm). Critical print size: 0.74–1.87 logMAR (N5-N64 at 10 cm).
Labib 2009 [29]	Cross sectional	*n* = 50 Group = low vision Age = 5–15 years (mean age 11 ± 2.6 y)	Test: Monocular near (Keeler’s reading chart) and distance (Landolt C) Distance: 25 cm Spacing: Not specified Response: Verbal	The near visual acuities ranged from A10 to A20, with the mean near acuity ± SD being A13.632 ± 3.17171.DVA ranged from 4/60 (0.06) to 6/24 (0.25), with mean distance visual acuity ± SD being 0.12 ± 0.12.
Boonstra 2012 [21]	Non-randomized controlled trial	*n* = 21 Group = low vision Age = 3 ½–6 years	Test: Binocular LEA near chart Distance: self-chosen distance, at 40 cm and at 3 metres Spacing: 0.5 and 1.0× letter size Response: Verbal	See Fig. 4.
Dekker 2012 [22]	Cross sectional	*n* = 62 Group = NV Age = 4–12 years	Test: Binocular LEA line and single at near and distance Distance: 0.3 and 3 metres Spacing: 0.5 and 1.0× letter size Response: Verbal	Distance vision crowding ratio (95% CI): 4–6y: 1.40 (0.88–2.22) 6–12: 1.31 (0.87–1.97) Near vision crowding ratio: 4–6y: 1.01 (0.55–1.86) 6–12y: 1.01 (0.72–1.42)
Huurneman 2012 [27]	Cross sectional	*n* = 58/*n* = 75 Group = low vision/NV Age = 4–8 years	Test: Binocular C-test and LEA line at near/C-test at distance Distance: 40 cm and 5 metres (if children had acuity < 20/125 distance was reduced to 2.5 metres at distance). Spacing: C-test: 2.6 and > =30; LEA line test 0.25, 0.5 and 1.0 × letter size Threshold: 60% (3/5) Response: Verbal	See Fig. 3.
Huurneman 2013 [28]	Non-randomized controlled trial	*n* = 45/*n* = 29 Group = low vision/NV Age = 4–9 years	Test: Binocular LEA version C-test + LEA line 50% at near; C-test Distance: 40 cm and 5 metres (viewing distance was reduced if DVA was < 20/125) Spacing: 2.6′ and ≥ 30′; 0.5× letter size Threshold: 60% (3/5) Response: Verbal	See Figs. 3 and 4.
Huang 2014 [26]	Cross sectional	*n* = 150 Group = NV Age = 3–5 years	Test: Binocular near-vision chart for children 3–5 years and the Chinese standard logarithmic near vision chart Distance: 25 cm Spacing: 1× letter size Response: Verbal	See Fig. 3.

*N refers to size of the letters, where one point is 0.35 mm (1/72 inch). ** A1 refers to 20/20 vision at 25 cm

### Impact method on NVA measured

Children aged 3 years and older can reply in a verbal manner and can grasp the concept of matching an answer to a stimulus. The response manner in the majority of studies is verbal naming or matching. Thirty years ago, a review on vision testing in 3–5 year old children reported that matching works reliably in a number of studies, while young children may refuse to give an adequate verbal response to testing [6]. The number of successful measurements was not reported in the majority of included studies, so no recommendations can be made with regards to the preferable response manner for children aged 4–7 years. In the nine quantitative studies that were included verbal response manners were used.

As can be seen in Fig. 3, there is a good correspondence between the Bailey-Lovie chart used to measure near visual acuity in 3–5 year olds and the Landolt C crowded chart in children with an average age of 5 years with NV. Another observation that can be made when looking at Fig. 3 is that Landolt C acuity and LEA acuities are in good agreement with each other. The near vision chart used by Dowdeswell was the same as the conventional chart used by Huang, but in the Dowdeswell study near visual acuity appears to be better. An explanation might be that the mean age of children in the latter study was higher.

In children aged 4–7 years, near visual acuity can be measured with crowded and uncrowded acuity charts. In three of the included papers uncrowded and crowded visual acuities were compared [21, 27, 28]. In children aged 4 to 7 years crowding, i.e. a deterioration of object recognition due to nearby flankers, is a normal phenomenon. When interpreting differences between uncrowded and crowded acuity scores, one should be aware that crowding decreases with age in children with NV. In children with NV, the difference between uncrowded and crowded visual acuity is 1–2 logMAR lines on the visual acuity chart (this entails crowding ratios between 1.25 and 1.6) [22, 27, 28]. In children with NV aged 6 years and older differences between uncrowded and crowded visual acuity of ≥ 3 logMAR lines are considered to be increased [22]. In children aged 10 or older crowding is practically absent, therefore a difference of 2 or more logMAR lines between crowded and uncrowded acuity is considered to be too large [33], because in adults with NV and older children ratios of 1.2 have been reported [34].

As can be seen in Table 4, the distance at which near visual acuity is assessed, differs considerably across studies. Data from the Boonstra study show that viewing distance does affect near visual acuity measures (Fig. 4). When children look at the chart at a self-chosen distance (which ranged from 5 to 20 cm), the acuity that is measured is ~0.15 logMAR lower than the acuity measured at 40 cm. The rationale behind the 40 cm rule is that accommodation is not expected to play a large role while at shorter distances it does [4]. Furthermore, it is difficult for the experimental leader to monitor viewing distance accurately if a child adopts a self-chosen viewing distance. In general, poorer acuity for near than distance vision has been reported before in subjects with NV and was attributed to errors in accommodation, for example accommodation lags (under-accommodation) or accommodation leads (over-accommodation) [35]. Near visual acuity (Snellen equivalent) is calculated by dividing the measurement distance in meters by the M-value. If this distance is reduced without necessity, the outcome is very likely to be lower and not representative for NVA.

### Method of choice

In children aged 4–7 years there is not such a clear golden standard as there is in 0–3 year olds. Near visual acuity can be assessed with several validated tests with comparable outcomes, for example the crowded Landolt C-test [31], the crowded LEA-version of the Landolt C-test [27] and the Bailey-Lovie chart [26]. Of these tests, the LEA-symbol is the preferred optotype, because children know the symbols, left-right confusion cannot affect measurements, symbols have been validated properly against Snellen E’s and Landolt C’s for size (LEA symbols have to be 1.5× larger than the E in order to result in the same VA), symbols have equal discriminability, and good test-retest reliability [36]. The appropriate response method is to let the child match the correct symbol or to ask the child to provide a verbal answer. Clinicians should keep in mind that viewing distance should be fixed, since small shifts in viewing distance can have a large impact on acuity, i.e. at 40 cm a 10 cm shift can cause a 25% change in visual acuity measures. Furthermore, inaccurate accommodation at smaller viewing distances is likely to exert a negative influence on the measured acuity. Finally, (near) visual acuity in children aged 4–7 years is highly affected by the presence of distractors in both children with NV and even more so in children with VI.

#### Near visual acuity assessments in 8–13 year olds

**Fig. 5:**
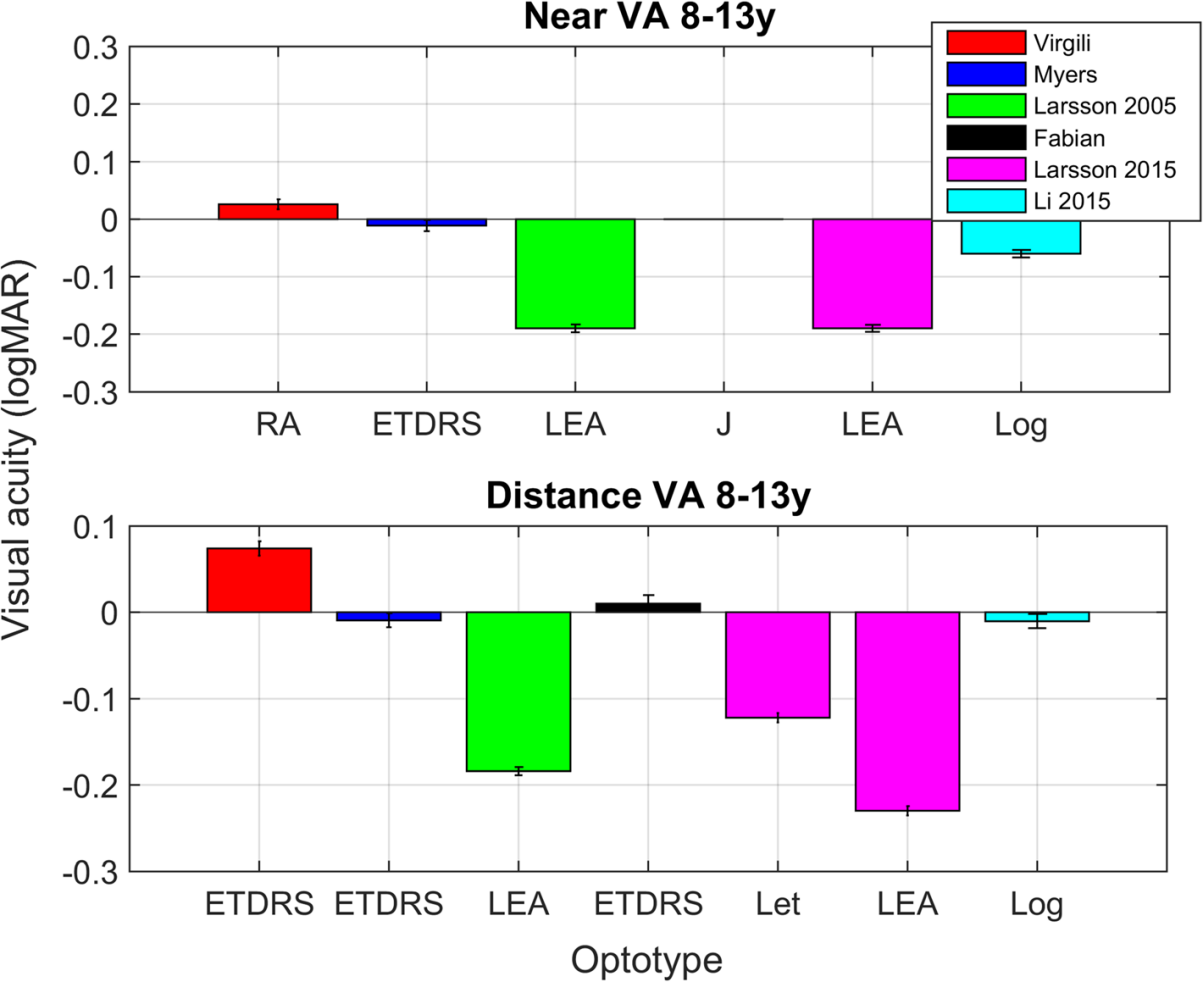
Near and distance visual acuity in 8–13 year olds with normal vision. As can be seen in this Figure, there was quite some variation in outcome. This variation can be due to differences in population characteristics across studies. Note that in the Larsson study, distance letter acuity was 0.11 logMAR poorer for letters than symbols. RA = reading acuity measured with the Italian version of MNREAD chart, ETDRS = ETDRS chart, LEA = Lea symbols, J = Jaeger chart, Log = LogMAR chart (type not specified). Error bars ± 1 sem

Ten studies were included in which near visual acuity was assessed in children aged 8 years and older [33, 37–45]. Table 5 presents the design, participant characteristics, method and outcome of the included studies. Figure 5 displays the results of 6 of the included studies in which means and standard deviations were provided.

**Table 5:** Type of study and outcome for quantitative studies on near visual acuity in 8-13 year olds

	Type of study	Number of participants, group (and age).	Method	Outcome
Peckham 1975 [43]	Cross sectional	*N* = 12772 Group = NV Age = 11 years	Test: Near vision (Sheridan Gardener test) and distance vision (Snellen chart) Distance: 25 cm and 6 m Optotype spacing: 1× letter size (edge-to-edge) Response: Verbal.	Distance vision: - Optimal vision (6/6 or better): 77.6% - Near optimal (6/9 or better): 10% - Moderate impairment (6/18-6/12): 7.1% - Severe impairment (≤6/24): 5.3% Near vision: - Optimal vision: 87.6% - Near optimal: 7% - Moderate impairment: 4% - Severe impairment:1.4%
Cummings 1996 [37]	Cross sectional	*N* = 1809 Group = NV Age = 8–10 years	Test: Not specified (near and distance vision) Distance: Not specified Optotype spacing: Not specified Response: Not specified	Normal vision (6/6): 69% Myopia: 24.3% Hypermetropia:0.7% Amblyopia: 1.5% Near vision problems: 12/1809 (<1%).
Myers 1999 [48]	Cross sectional	*N* = 106 Group = NV Age = 10 years	Test: ETDRS chart (near and distance) Distance: 40 cm and 4 m Optotype spacing: Not specified Response: Verbal	See Fig. 5.
Wolffsohn 2000 [45]	Cross sectional	*N* = 53 Group = VI Age = 9–91 (median age 80 years)	Test: Practical near acuity card (PNAC) and Bailey-Lovie near and distance chart Distance: 25 cm (near). Distance Bailey-Lovie unspecified. Optotype spacing: default Times new roman spacing (approx. 0.1× letter size) and 1× letter size Response: Verbal.	Mean DVA was 0.91 ± 0.04 logMAR. Good correlation between distance VA and PNAC (*r* = 0.74). No differences between PNAC and near Bailey-Lovie chart measures (*r* = 0.97).
Virgili 2004 [44]	Cross sectional	*N* = 116 Group = NV Age = 6–12 years	Test: Italian version MNREAD, distance vision ETDRS Distance: 40 cm, ETDRS distance not specified Optotype spacing: reading setting (approx. ×1.1 letter size) Response: Verbal.	See Fig. 5.
Larsson 2005 [33]	Cross sectional	*N* = 217 Group = NV Age = 10 years	Test: LEA chart (near and distance) Distance: 40 cm and 3 m Optotype spacing: not specified Response: Verbal.	See Fig. 5.
Hanson 2006 [39]	Cross sectional	*N* = 26 Group = VI Age = 10–50 years	Test: S-charts at 40 cm and 3.75 m, Bailey-Lovie at distance (6 m) and ETDRS at near (preferred working distance). Distance: 40 cm, 3.75 m and 6 m. Optotype spacing: 1× letter size Response: Verbal	No consistent differences between near and distance VA’s.
Fabian 2013 [38]	Cross sectional	*N* = 66 Group = NV Age = mean age 9 years	Test: Jaeger (near) and ETDRS (distance) Distance: not specified. Optotype spacing: not specified Response: Verbal.	See Fig. 5. All children with NV had a J1* score for near vision.
Larsson 2015 [40]	Cross sectional	*N* = 217 Group = NV Age = 10 years	Test: LEA test (near and distance), linear logMAR chart (distance), LEA single optotypes (3 m) Distance: 40 cm and 3 or 4 meter Optotype spacing: not specified Response: Verbal.	See Fig. 5.
Li 2015 [41]	Cross sectional	*N* = 190 Group = NV Age = 10–14 years	Test: logMAR visual acuity chart Distance: 40 cm and 4 meter Optotype spacing: not specified Response: Verbal.	See Fig. 5.

*J1-J20 is sized 0.5-19.5 mm (J1 = 20/20 at 34.4 cm

### Impact method on VA measured

In the 70’s and 80’s the Sheridan Gardener test was often used to measure near visual acuity in school aged children and the Snellen chart was used to measure distance visual acuity [32, 43]. Nowadays, the ETDRS chart [38, 39, 42, 44], the Bailey-Lovie letter chart [45] and LEA charts [33, 40] are more frequently used to assess near visual acuity in school-aged children. The ETDRS and Bailey-Lovie letter chart can be used in children that can read letters. As can be seen in Fig. 5, the method that is used to measure near visual acuity has a profound effect on the outcome. The two Larsson studies used LEA symbols to measure acuity in 10 year olds and report much better acuities than the other four studies in which letters were used to measure acuity. One study compared distance visual acuity for letters with distance visual acuity for LEA symbols and found a difference between these two methods of almost 1 logMAR line [40]. Viewing distance was 40 cm for the majority of studies (6/8 studies where viewing distance was reported). In two studies a viewing distance of 25 cm was used [43, 45].

Two studies were included on near visual acuity assessments in subjects with VI [39, 45]. The goal of the Wolffsohn study was to develop a reading chart that could be used to assess near vision in a quick and accurate manner in low vision patients [45]. The authors emphasize that near visual acuity measurement is an important clinical measurement as many visual demanding tasks are performed close to the eyes, especially by patients with low vision. In addition, certain ocular disorders can have a differential effect on near and distance visual acuity. The drawbacks of other near vision charts that are mentioned by the authors are that reading charts are often slow to establish a near acuity threshold (the example being mentioned is the Bailey-Lovie chart), especially in those with reduced vision, poor language or weaker cognitive skills, and that they often lack realism to commonly performed tasks such as reading the paper or telephone book. A Practical Near Acuity Chart (PNAC) was developed to measure near visual acuity as accurately as the available charts in patients with VI, but in less time. The PNAC incorporates 8 important design features: large (N80 or 1.6 logMAR at the prescribed 25 cm reading distance) to small print size (N5 or 0.4 logMAR at 25 cm reading distance), regular progressions between lines (0.1 logMAR), an equal number of words on each row, one three-letter word one four-letter word and one five-letter word per row, the use of word sequences that are related, easy to recognize for 9-year old children, Times New Roman font (common font), and paragraphs of the most commonly used print sizes on the backside to determine reading speed and fluency. The PNAC near acuity thresholds and Bailey-Lovie near acuity thresholds did not differ, while test duration was longer for the Bailey-Lovie chart (32 ± 2 s versus 76 ± 4 s).

The second study in which near visual acuity was measured in children and adults with VI due to infantile nystagmus aimed to assess differences in near and distance visual acuity [39]. This was done because there was controversy in the literature about the effect of viewing distance on visual acuity in individuals with infantile nystagmus. It was hypothesized that, in patients with infantile nystagmus, near visual acuity could be better because of a dampening of the nystagmus intensity at near compared to distance viewing. This dampening of nystagmus was indeed observed, but there were no consistent relations between the dampening of nystagmus and an increase in acuity suggesting that visual acuity in subjects with infantile nystagmus appears to be limited more by sensory than oculomotor deficits.

### Method of choice

The ETDRS was the most frequently used chart to measure near vision in children aged 8 years or older. There are clinically relevant differences in visual acuity measured with letters and visual acuity measured with symbols (differences appear to be ~0.1–0.2 logMAR). Therefore, clinicians should report the chart that was used to measure acuity. The relatively lower acuity for ETDRS letter charts might be caused by a relatively low guess rate (10%), and the use of SLOAN letters, which are more difficult to discriminate than resolution or symbol optotypes. The benefit of using letters instead of symbols is that letter recognition lies closer to reading ability than symbol recognition.

## Discussion

The general goal of this review was to provide guidelines for near visual acuity measurement for 0–13 year old children with normal vision and low vision. The most frequently used method to measure visual acuity in 0–36 month old children was the TAC. There was no clear method of choice for the intermediate age group, the 4–7 year old children. For the 8–13 year old children, the ETDRS was the most frequently used chart. Nine studies were included that measured near and distance visual acuities (reporting means and standard deviations), but there were no systematic difference between these measures. Standard deviations were not larger for near than distance visual acuities, suggesting that reliability of these measures is not weaker than reliability of distance visual acuities when measured with a standardized routine. This review brings forth several insights with regards to four topics: (i) the impact of threshold choice on vision outcome, (ii) the sensitivity of charts to detect refraction errors or crowding, (iii) the impact of viewing distance during near vision assessments, and (iv) the impact of optotype choice on the measured visual acuity.

### Threshold choice: the need for standardization

For 0–36 month old children, the TAC procedure is the clear golden standard. During the development of the TAC in the early ‘80s, there were studies that compared outcomes collected with different thresholds (for example 60% and 75% [12], or 55% and 70% [14]). In psychophysics, the threshold is often defined as the ‘halfway up point’ that lies halfway between the guess rate (γ) and 100% correct rate [46]. So, when applying this psychophysics threshold rule to the TAC, which in essence is a 2 alternative forced choice task (2AFC), a 75% correct threshold should be used. However, the 70% threshold collected with forced preferential looking procedure in 3-month olds [14] lies well below the lower border of the norms collected by Salomao et al. [17]. The same trend was observed when comparing the 75% FPL thresholds collected by Mayer et al. [12] with the lower bounds collected by Salomao et al. [17]. Salomao et al. used a modified staircase procedure to measure grating acuity with the TAC, which entails that cards were presented from lower to higher spatial frequencies in one octave steps up to the threshold region and then 0.5 octave steps around the threshold. Testing continued until two consecutive staircase reversals occurred. The VA threshold was defined as the spatial frequency that received two positive responses. If this could not be done in six trials (counting from the first negative response), threshold was defined as the highest spatial frequency that received the greatest number of positive responses. In the 4–7 year olds, only 4 studies reported the scoring procedure that was used. The Hohmann study in which the C-test was validated used an 88–94% threshold, the Huurneman studies used a 60% threshold, and Lovie-Kitchin scored the acuity per letter (5 letters per line 0.02 logMAR per letter on a row). Studies in the oldest age group did not report which thresholds were used. Considering the large differences in outcome between studies, threshold choice might have affected vision outcome; using a high threshold (75% or higher) can result in lower acuities than using a lower threshold (50–60%). Standardization is needed to enable comparison between different acuity scores. For a 4AFC task, such as the C-test or the LEA symbols, a 62.5% threshold lying halfway between the guess rate and 100% correct would be preferable [46].

### Sensitivity for detection of near vision problems: use optotype charts

For children aged 3 years and older it is preferable to use charts that are more sensitive than the TAC [18]. The Landolt C test is more sensitive in detecting uncorrected refractive errors than the TAC and results in significantly lower acuity estimates [18]. The explanations that the authors offer for the lower Landolt C versus TAC-scores are: task complexity, acuity gradation (inter-card intervals TAC correspond to 2–3 acuity lines of the C-test), and the higher sensitivity of the C-test to detect uncorrected refractive errors. In addition, differences in testing procedure might also affect measurements: the uncrowded C-test offers 6 optotypes per row and the authors allowed one mistake per row [18]. This means that thresholds for the C-test were higher than for the TAC test, which could in itself result in poorer acuity estimates measured with the C-test compared to the TAC acuity. In addition, the TAC and Landolt C test are both examples of tests measuring resolution acuity, but the Landolt C ring has higher sensitivity for detecting uncorrected refractive errors than the TAC and in case of the Landolt C matching is possible which provides a more reliable response. Explanations for this could be that the TAC stimulus is larger (12.5 × 12.5 cm) than the C-test stimulus. Lower acuity estimates collected with the C-test than the TAC might also be explained by: (i) higher attentional demands, (ii) higher oculomotor demands, and (iii) the contour interaction effects present in the C-test (when using the crowded chart version).

As was mentioned in an earlier review, separate norm scores should be used when interpreting uncrowded and crowded acuity scores [6]. For children aged 6–10 years, crowding ratios of 2 and higher can be considered as increased [22]. In children aged 10 and older, crowding ratios of 1.5 can be seen as increased [40]. Studies report different findings with regard to the influence of viewing distance on crowding ratios. Dekker et al. reported lower near crowding ratios than distance crowding ratios, but did not have an explanation as for why this occurred. Huurneman et al. consistently found higher crowding ratios at near than at distance [27, 28]. These studies use different methods, but this difference does not offer an explanation for the observed finding. More research is needed to find out what mechanisms underlie differences between near and distance crowding ratios. In order to be able to detect vision problems due to crowding, it is of crucial importance to use sensitive screening charts. In general, sensitivity of visual screening tests can be improved by using flankers that are more tightly spaced and letter like with an optimal spacing of ~1.13× the optotype size [47].

During general school screenings, near vision is not a mandatory part of vision screenings, since there is evidence that poor distance vision has a higher prevalence than poor near vision [37, 43]. Therefore, near vision assessments do not seem to add value to general school screenings in children with normal vision and should be done when a child has reduced vision or experiences problems when reading or when doing near work.

### Viewing distance and near visual acuity

Measuring visual acuity at a viewing distance chosen by the child can cause serious underestimations of near visual acuity in children (0.16 logMAR or 1.6 lines difference on a vision chart) [21]. An explanation for this is that under- or over accommodation of the lens can result in poorer acuities. In addition, it is difficult for the test leader to monitor viewing distance when the child is moving. There is substantial variability in viewing distances adopted across the studies that were included in this review. In older children, the majority of studies use a viewing distance of 40 cm. However, for the 3–6 year olds, viewing distance is as often 25 or 30 cm as it is 40 cm. With the exception of one study [21], there are no other studies systematically evaluating the effect of viewing distance with the same set of subjects, and therefore comparison between studies is not possible. Differences in near vision scores of 5 year olds for crowded optotypes [21, 26, 27] suggest that the 25 cm viewing distance could result in somewhat lower acuities, but since different children were tested across studies this comparison cannot be made. Viewing distance should be controlled for and, ideally, should be fixed and at 40 cm for near visual acuity because of the influence of accommodation, and in order to allow comparisons between studies. Experiments by Huurneman et al. show that children aged 4 years and older can very well respect the distance of 40 cm during near vision measurement [27].

### Optotype choice: letter versus symbol

Optotype choice has an impact on near visual acuities measured with letter scores resulting in lower acuity estimates than charts with symbols. This difference between acuity measured with letters and acuity measured with symbols was consistent across several studies and several age groups. Visual acuities measured with the 4AFC C-test and LEA symbols correspond well with each other [27, 28]. The ETDRS test presents 5 SLOAN letters per row with equal legibility, spacing between letters and rows is consistent; and size varies with (0.1) logarithmic intervals between lines. A limitation of the ETDRS chart is that letters cannot be read in countries where Roman characters are not used.

### Near and distance visual acuity

There were no consistent differences between distance and near visual acuities. Myers et al. did not find a difference between near and distance ETDRS acuity in children with normal vision [48]. Larsson et al. found no differences between near and distance VA in the first study, but did report better distance than near VA in the last study [33, 40]. The Fabian study reported no differences between distance and near [38]. In contrast with the Larsson studies [33, 40], Li et al. reported better near than distance VA [41]. Huurneman et al. found no clear near distance differences in children with VI [27, 28]. Boonstra et al. found better distance than near visual acuity in 3 ½–6 year old children with VI [21]. Finally, Dowdeswell et al., reported better near than distance visual acuity in children with NV while using the Bailey-Lovie chart at 0.3 and 6 m [23]. Collectively, the results of the included studies indicate that there no evidence for robust systematic differences between near and distance visual acuities in children with normal vision and children with low vision as long as the 40 cm distance at near is maintained during measurement.

## Conclusions

This review shows that for 0–36 month old children there is a golden standard for near visual acuity assessments: the Teller Acuity Cards (TAC). In 4–7 year olds, we recommend use of the LEA-chart at 40 cm because it has been validated properly and can be used in preliterate children without inducing left-right confusion. In 8–13 year olds, the ETDRS seems to be the preferable chart. The following guidelines for clinical practice can be extracted from this review: (i) visual acuity should be measured with the most sensitive chart that is validated for a variety of subjects, (ii) the choice for a specific chart should be based on developmental age, (iii) different norms should be used for crowded and uncrowded acuities and norm values applied to estimate the age-dependant amount of crowding, (iv) for very young children, low thresholds or maximum correct response should be used, and (v) viewing distance and thresholds should be standardized (preferably a viewing distance of 40 cm).

## Abbreviations

ETRDS: Early Treatment Diabetic Retinopathy Study
FPL: Forced preferential looking
LogMAR: The logarithm of the minimum angle of resolution
NV: Normal vision
OKN: Optokinetic nystagmus
PNAC: Practical Near Acuity Card
TAC: Teller Acuity Card
VI: Visual impairment
